# Supplementary figures and images for: Dual RNA-seq reveals transcriptome changes during Fusarium virguliforme-Trichoderma afroharzianum interactions
Source: PLoS One. 2025 Jan 24;20(1):e0310850. doi: 10.1371/journal.pone.0310850 (PMC11761082; doi:10.1371/journal.pone.0310850)

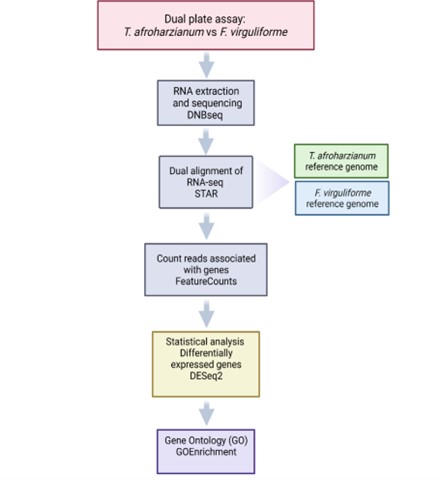

Supplement: S1 Fig — (JPG) [file pone.0310850.s001.jpg]

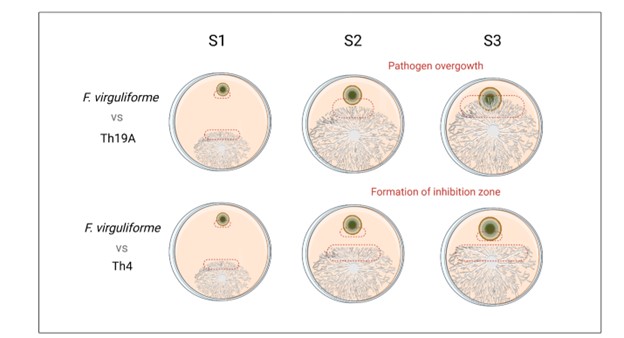

Supplement: S2 Fig — Red dotted areas represent areas where fungal mycelia were collected for RNA isolation and sequencing. S1 = first stage of interaction before any physical contact between fungi; S2 = second stage of interaction where Th19A touches Fv colony and Th4 starts formation of inhibition zone; S3 = third stage of interaction where Th19A overgrows Fv and complete inhibition zone is formed between Fv and Th4. (JPG) [file pone.0310850.s002.jpg]

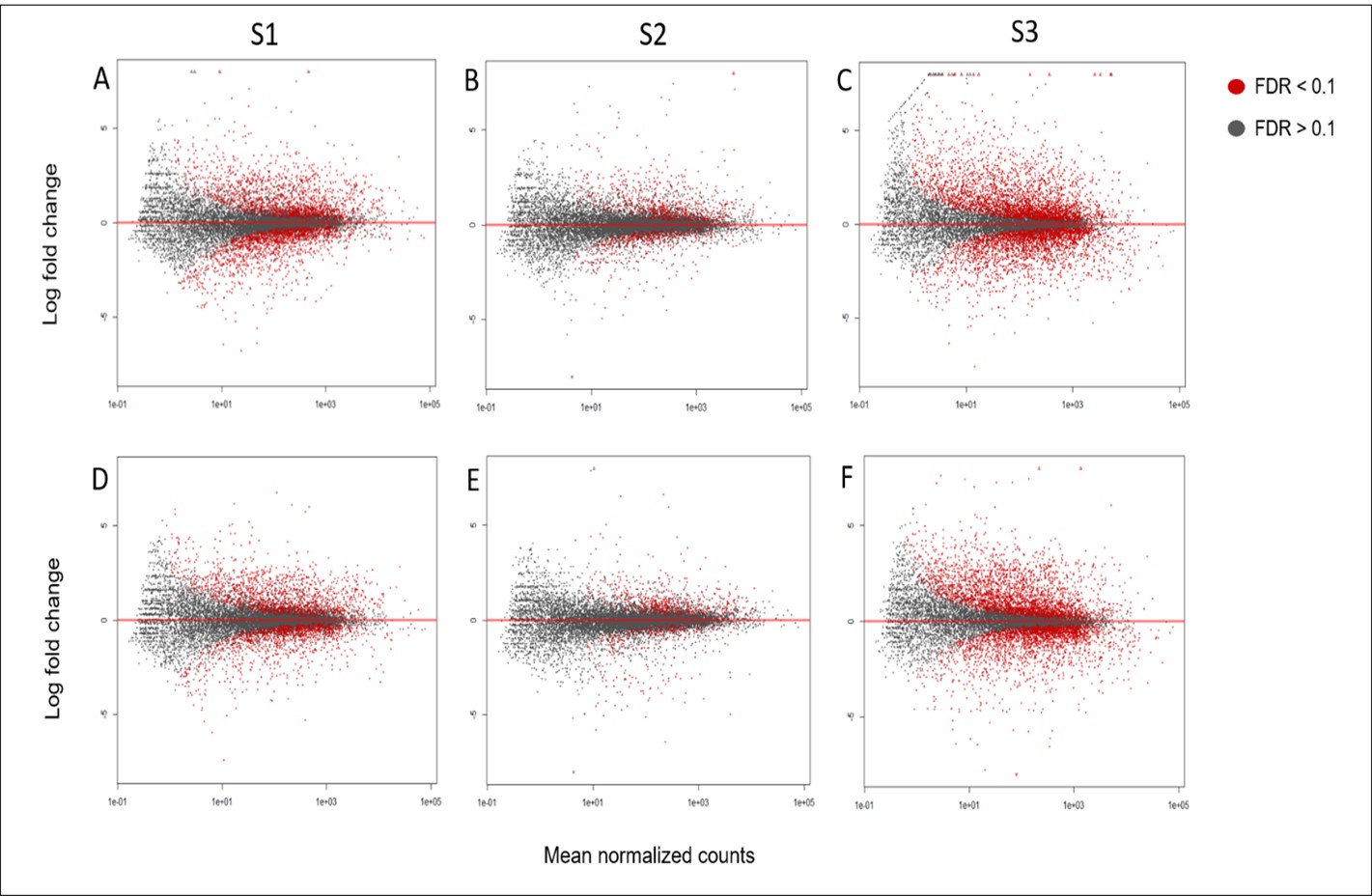

Supplement: S3 Fig — MA-plots illustrate the log fold change of the Fv differentially expressed genes (red). S1 = before physical contact, S2 = at contact (Th19A) or beginning of inhibition zone (Th4), S3 = overgrowth (Th19A) or complete inhibition zone (Th4). Differentially expressed genes were determined using DESeq2 and normalized against the control (Fv growing alone). (JPG) [file pone.0310850.s003.jpg]

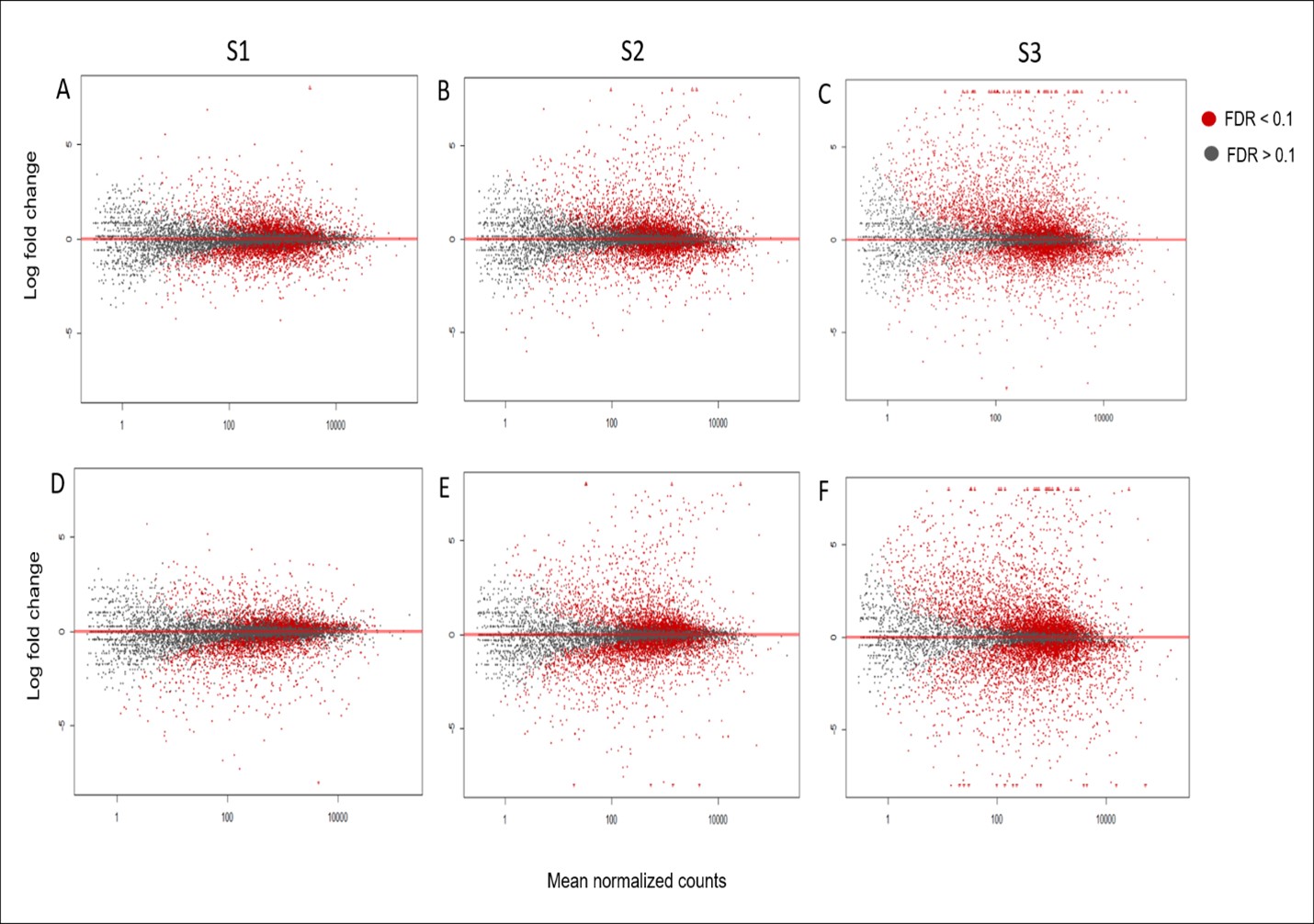

Supplement: S4 Fig — MA-plots illustrate the log fold change of the Th19A and Th4 differentially expressed genes (DEGs) in red. S1 = before physical contact, S2 = at contact for or beginning of inhibition zone formation, S3 = overgrowth or complete inhibition zone. DEGs were determined using DESeq2 (False discovery rate (FDR) < 0.1) and normalized against the control (Th19A and Th4 growing alone). (JPG) [file pone.0310850.s004.jpg]
